# The Learning Curve of Reverdin–Isham and Akin Percutaneous Osteotomies for Hallux Valgus Correction: A Bayesian Approach

**DOI:** 10.3390/jcm14061921

**Published:** 2025-03-12

**Authors:** Carlo Biz, Elisa Belluzzi, Alberto Crimì, Giovanni Sciarretta, Elena Bortolato, Pietro Ruggieri

**Affiliations:** 1 Orthopedics and Orthopedic Oncology, Department of Surgery, Oncology and Gastroenterology DiSCOG, University-Hospital of Padova, Via Giustiniani 3, 35128 Padova, Italy; carlo.biz@unipd.it (C.B.); alberto.crimi@aopd.veneto.it (A.C.); giovanni.sciarretta@studenti.unipd.it (G.S.); pietro.ruggieri@unipd.it (P.R.); 2 Centre for Mechanics of Biological Materials, University of Padova, 35131 Padova, Italy; 3 Musculoskeletal Pathology and Oncology Laboratory, Department of Surgery, Oncology and Gastroenterology (DiSCOG), University of Padova, Via Giustiniani 3, 35128 Padova, Italy; 4 Department of Business and Economics, Universitat Pompeu Fabra, 08005 Barcelona, Spain; elena.bortolato@bse.eu; 5 Data Science Center, Barcelona School of Economics, 08005 Barcelona, Spain

**Keywords:** hallux valgus, learning curve, Reverdin–Isham, akin osteotomy, Bayesian change point model

## Abstract

**Background/Objectives**: Assessing the learning curve is essential for surgical techniques that require precision and technical adaptation. Although modified Reverdin–Isham and Akin percutaneous osteotomies (RIAOs) are well-established procedures for the treatment of hallux valgus (HV), their percutaneous nature and specific technical demands justify the evaluation of the learning curve. Therefore, this study aimed to assess the learning curve of RIAOs for the HV correction, using for the first time a Bayesian approach. **Methods**: Modified RIAOs were applied to treat mild-to-moderate HV in patients who were prospectively enrolled. The hallux valgus angle (HVA), inter-metatarsal angle (IMA), distal metatarsal articular angle (DMAA) and tibial sesamoid position were assessed. Clinical outcomes were evaluated with the American Orthopaedic Foot & Ankle Society (AOFAS) Scale, Visual Analog Scale (VAS) and Numerical Rating Scale (NRS). Surgery and fluoroscopy times were recorded. To evaluate the learning curve, a Bayesian analysis using a change point model was performed. **Results**: Analysis of 142 patients revealed three distinct phases in the learning curve, with a plateau reached after 112 procedures. Over time, the mean operation duration decreased from 55 to 27 min, and fluoroscopy time decreased from 60 to 28 s. **Conclusions**: A flexible change point model was used to model a learning curve, guaranteeing a robust interpretation of the data. The correction of the HV angles showed similar results in the three phases of the curve, demonstrating that the surgeon achieved positive results from the beginning of the surgery.

## 1. Introduction

Learning curve analysis is pivotal to understanding the outcome, complication rate, reoperation rate, reliability and improvement of a new surgical technique [1]. Today, learning curves are also important in education, research and randomized controlled trial design [2]. As shown in other fields, the learning curve of a surgical technique is normally divided into two phases: a “learning phase”, where outcomes can be suboptimal, and learning is rapid, and a “plateau phase” where proficiency has been reached. In this way, it is possible to determine the best mentorship time for a new surgeon and the minimal number of cases a surgeon should perform with a tutor before starting to operate autonomously and safely [3,4]. The required proficiency can be measured by numerous parameters depending on the specifics of the procedure in question [5].

In the last two decades, minimally invasive surgery (MIS) for hallux valgus (HV) correction has increased in popularity, and the learning curve analysis was used to demonstrate the safety of the techniques and their reproducibility [5,6,7]. Distal metatarsal osteotomies are the most used surgical techniques for HV treatment, and among these, Reverdin osteotomy was the first to be described in 1881 as a subcapital closing wedge osteotomy [8]. Currently, it is considered a first-generation percutaneous bunion repair [9]. The technique was further modified in 1970 by Funk and Wells [10], removing a medial wedge of bone from the first metatarsal to correct the metatarsophalangeal angle. At the beginning of the new century, this osteotomy, previously modified by Isham [9,11], was further improved by De Prado who proposed a percutaneous procedure based on the association of a modified distal metatarsal osteotomy (Reverdin–Isham technique) with a phalangeal osteotomy (Akin procedure) and tenotomy of the abductor hallucis tendon [12]. During the first two decades of the 21st century, this method was rapidly becoming popular, particularly in Europe, as it is quick to perform, allows 1-day hospitalization, decreases postoperative morbidity as well as recovery and rehabilitation times and primarily because it was better accepted by patients [5]. More recently, however, it has been increasingly replaced by second- and third-generation MIS [5,7,13].

Despite being a first-generation percutaneous technique, previous studies showed that the Reverdin–Isham and Akin percutaneous osteotomies (RIAOs) in combination with lateral soft-tissue release (LR) are safe, effective and reliable procedures for the correction of mild-to-moderate symptomatic HV [11,14,15]. The main advantage appears to be the preservation of soft tissues, which leads to reduced recovery and rehabilitation times, decreasing the morbidity associated with the disease process and the operative intervention. However, it should be performed by experienced surgeons with knowledge of open, minimally invasive and percutaneous techniques [16], as several aspects make this procedure challenging. First, it is defined by six main steps: exostosectomy, Reverdin–Isham osteotomy, adductor hallucis tendon tenotomy, lateral capsulotomy, Akin osteotomy and bandaging. Even though these steps involve small incisions (1–3 mm), each requires a meticulous and detailed knowledge of the anatomical relationships within the surgical field to avoid injury to the forefoot structures [17]. Second, the technique requires specific tools (dedicated knife and scalpels, burrs of different sizes and forms and modular power driver for MIS), which must be well known and handled after appropriate training. Third, since the RIAOs are not complete osteotomies and do not involve fixation, possible errors in not preserving the lateral cortex can lead to osteotomy instability, delayed healing, non-union, malalignment and loss of correction [18]. Finally, correct bandaging is essential to maintain the osteotomies in the proper position: errors in its packaging technique during the postoperative period lead to failures.

Limited data exist on the exact nature of the learning curve in MIS for HV and its impact on surgeon training, complication rates and patient outcomes [5,19]. To date, no study has examined the impact of the learning curve for the percutaneous RIAOs associated with LR for HV correction.

Thus, this study aimed, first, to assess the learning curve of a single surgeon to understand when the plateau phase was reached in this percutaneous technique and, second, to verify what the improvements in surgical time, fluoroscopy time, postoperative complication rate, reoperation rate, patient outcomes and patient satisfaction after surgery are. We hypothesized that there was an improvement in the various parameters evaluated such that it does not justify abandoning this procedure in surgical practice after its widespread diffusion in the early 2000s.

## 2. Materials and Methods

### 2.1. Patients

Patients consecutively treated for symptomatic HV from 2010 to 2021 were prospectively enrolled at our institution. Inclusion criteria were mild or moderate HV deformity treated by the same surgeon using the percutaneous RIAOs associated with LR as described by De Prado [12]. The surgeon’s background was 5 years of experience in foot and ankle surgery and participation in 3 MIS cadaver labs (MIFAS by Grecmip) before starting the first cases enrolled in this study.

Patients treated with other techniques or who underwent correction of other foot deformities correlated with HV in the same surgical session were excluded from the study. Other exclusion criteria were congenital deformities of the foot, hallux rigidus, personal history of traumatic or surgical injuries to the affected foot and ankle and the presence of rheumatic, dysmetabolic, neurological, infective or psychiatric pathologies. The study was approved by the Local Ethics Committee of Hospital-University of Padova (protocol code 4064/AO/17). It was conducted according to good clinical practice guidelines and the ethical standards of the 1964 Declaration of Helsinki as revised in 2013. All patients received a thorough explanation of the risks and benefits of inclusion and gave their written informed consent to be included in the study.

### 2.2. Surgery and Postoperative Protocol

The Percutaneous RIAOs were performed under control as described in previous studies [9,11,14,19,20,21]. Through a 3 to 5 mm incision on the medial border of the first metatarsal head, the exostosis was removed from the first metatarsal head (exostosectomy) using a cylindrical burr. Through the same incision, a 12 mm Shannon Isham burr was used to perform a Reverdin–Isham osteotomy at the junction of the metaphysis and epiphysis (distal metatarsal osteotomy). A wedge burr was then used to create a wedge with a medially oriented base. The closure of the wedge modified the orientation of the articular surface, normalizing the distal metatarsal articular angle (DMMA). Damage to the dorsomedial cutaneous nerve of the hallux was prevented by the position of the incision and the clock rule [22].

Using a second small longitudinal skin incision on the first web space, a percutaneous release of the adductor hallucis tendon and the lateral portion of the joint capsule was carried out (LR). The last incision was a 3 to 5 mm incision made on the medial surface of the base of the proximal phalanx of the hallux to perform Akin osteotomy. Because fixation was not performed, careful post-surgical bandaging was essential to maintain the correction. Full weight bearing was allowed immediately after surgery using a rigid flat-soled orthopaedic shoe for a month. The postoperative follow-up was fixed weekly for 4 weeks, with replacement of the bandage every visit and weight-bearing X-rays at 4 weeks from surgery. Patients used a silicone spacer to keep the hallux in the correct position for 3 months, and they were advised to avoid overload of the forefoot for 6 months after surgery.

**Figure 1:**
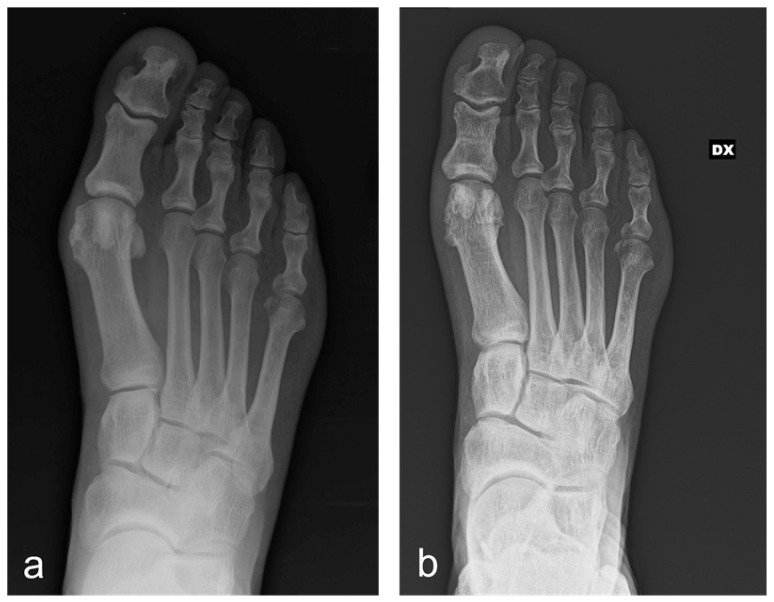
A 71-year-old man having undergone percutaneous Reverdin–Isham osteotomy, lateral release and Akin osteotomy for moderate HV correction: antero-posterior radiographic images at preoperative period (**a**) and 3-month follow-up (**b**), showing the complete healing of the osteotomies and the correction of the deformity. (DX: right foot).

According to our institutional protocol, routine X-rays in anterior-posterior, latero-lateral and sesamoid views were obtained before surgery, immediately after surgery, at 1 month and 3 months post-surgery (all weight-bearing except for the immediate postoperative one) (Figure 1).

### 2.3. Radiological and Clinical Evaluation

The radiological and clinical analyses were carried out by two independent investigators, the junior authors (A.C. and G.S.), who were not directly involved in the patients’ operative treatment. The hallux valgus angle (HVA), the inter-metatarsal angle (IMA), DMAA and tibial sesamoid position (TSP) were evaluated for each patient on weight-bearing anterior-posterior, latero-lateral and sesamoid X-ray views at preoperative period and at 3-month follow-up. These radiographic parameters were analyzed in a standardized manner using electronically computer-assisted measurements for weight-bearing radiographs. After a period of training in HV measurement techniques by the senior author (C.B.), the two reviewers who performed radiographic evaluation used a digital workstation and software (MedStation 4.9.907.0 program X-ray database of our hospital, Exprivia Healthcare IT Srl, Bari, Italy) to minimize bias during the measurements [23]. This software allows the retrieval of those electronically computer-assisted measurements previously described from weight-bearing radiographs. Intra-reader and inter-reader reliability were assessed using the Intraclass Correlation Coefficient (ICC), with values of 0.83 and 0.81, respectively.

The severity of HV was defined according to the Mann and Coughlin [24] parameters (only mild and moderate HV was considered eligible for the surgical technique):

- Mild HV (grade 1): HVA ≤ 20°, IMA ≤ 11°, less than 50% subluxation of the medial sesamoid;
- Moderate HV (grade 2): HVA between 20° and 40°, IMA between 11° and 16°, 50 to 75% subluxation of tibial sesamoid.

Clinical data collected for every patient included the following: complete clinical history; demographic characteristics (gender, age at the time of surgery, affected side); American Orthopaedic Foot & Ankle Society Hallux Metatarsophalangeal-Interphalangeal Scale (AOFAS MTP-IP) [25,26] before surgery and at the clinical evaluations 3, 12 and 24 months after surgery; patient satisfaction using the Visual Analog Scale (VAS) at 24-month after surgery with a score ranging from 1 to 10; pain was rated before surgery, after surgery and at follow-up visits by the patients with the Numerical Rating Scale (NRS). Time of surgery and fluoroscopy use was recorded for each patient as well as complications.

### 2.4. Statistical Analysis

To establish if there exists stabilization at the end of the learning process, we considered a Bayesian change point model (see, e.g., Eckley et al., 2005) [27], a statistical method to identify changes, shifts and phases in a process over time. In the present case, we examined the number of patients treated by the surgeon and characterized these periods. The periods to be identified were three, indicated as 1, 2 and 3, and were separated by two “changepoints-endpoints”, $\tau_{1}$ and $\tau_{2}$, that are unknown and had to be determined by analyzing the data together with other regression parameters.

A Bayesian approach for estimating the change point model was preferred because the Bayesian posterior distribution naturally captures the uncertainties in all parameters, which are interdependent. Specifically, the uncertainty in the change point location influences uncertainties in other parameters, such as the learning rate and variability. Conversely, classical inferential procedures (confidence intervals) for all such parameters would depend on the selection of a single best-fit change point, without carrying the uncertainty, which can lead to overconfidence.

A priori, it was only assumed that the first shift could take place before 40 patients ($\tau_{1}$ < 40), while the second could take place between 100 and 140 ($100<\tau_{2}<140)$. Within each period, the model is a normal regression model with period-specific coefficients.

Denoting with $y_{tj}$ the operational time at the time of the *t*-th patient and in the *j*-th time window, the model was

$$y_{tj}=\beta^{j}{x^{j}}_{t}+\gamma^{j}\left(t-\tau^{\left\{j-1\right\}}\right)+\epsilon_{tj},\;\mathrm{if}\;\tau^{j-1}<t<\tau^{j},\;\mathrm{with}\;j=1,2,3,\;\tau^{0}=0,\tau^{3}=140.$$

Here, $\gamma^{j}$ indicates the learning rate. We were interested in verifying that $\gamma^{3}=0,$ meaning that the rate is 0 at the third period and the surgeon is no longer learning.

$\beta^{j}{x^{j}}_{t}$ is the linear predictor that includes regression coefficients specific for the *j*-th time window (The intercept is specific for the time window and the dependence on the fluoroscopy time).

It was assumed that the error terms were independent, $\epsilon_{t}^{j}∼N\left(0,\sigma^{2j}\right),$ with variance $\sigma^{2j}$ for the corresponding time window, meaning different variability in the operational time per each time window.

In the linear predictor, an intercept $\beta^{j0}$ was included, representing the operational time at the beginning of the phase. Diffuse low-informative prior distributions for the parameters, $\beta^{j}∼\;N\left(0,5\right)$ and $\gamma^{j},\sigma^{2j\;}∼\mathrm{Gamma}\left(1.2,0.05\right),$ were assumed. To estimate the model, Markov Chain Monte Carlo sampling with the Metropolis within the Gibbs algorithm was employed [28].

For all preliminary analyses, a *p*-value less than or equal to 0.05 was considered statistically significant. Spearman’s rank correlation test was performed to assess correlations between all variables and patient order. All statistical analyses were performed using R version 4.4.1 (2024-06-14) [29].

## 3. Results

**Figure 2:**
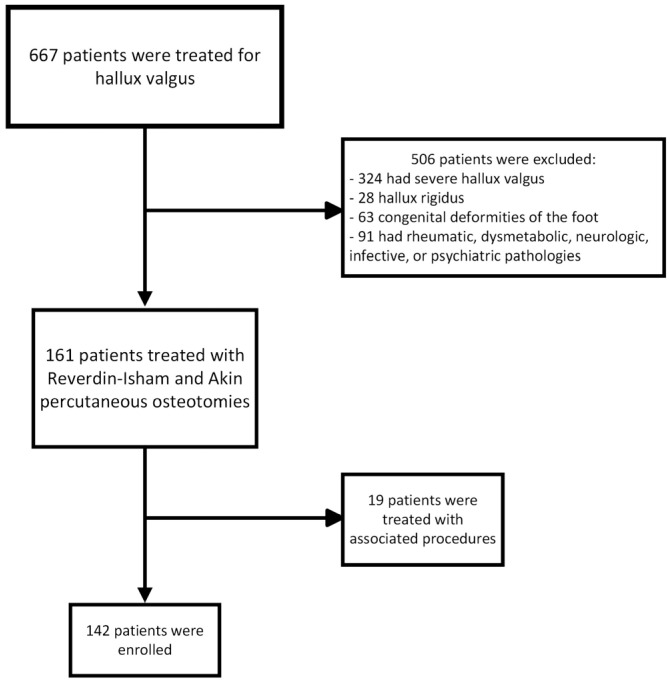
Flow chart of patient selection.

A total of 142 patients were enrolled in this study, 127 females (89.4%) and 15 males (10.6%) (Figure 2).

The average age of the patients was 51.1 ± 14.8 years. A total of 127 (89.4%) patients were classified as moderate HV and 15 (10.6%) as mild HV.

The surgical outcome was evaluated with standing X-rays pre-op, post-op and 3 months after surgery, measuring HVA, IMA and DMAA.

The mean difference in the angles and NRS at the baseline and 3 months after surgery was then calculated (*p* < 0.0001) (Table 1).

All 142 patients were administered the AOFAS Scale preoperatively, at 3 months, at 1 year and at 2 years after surgery (49.6 ± 9.0, 69.2 ± 9.0, 79.25 ± 10.9 and 84.6 ± 12.0, respectively, *p* < 0.001). The results showed an improvement in the patients treated with this technique; the NRS improved from 6.8 ± 1.3 preoperatively to 1.3 ± 1.3 at 24 months follow-up (*p* < 0.001). VAS satisfaction was reported to be 7.5 ± 1.9 at the last follow-up.

**Figure 3:**
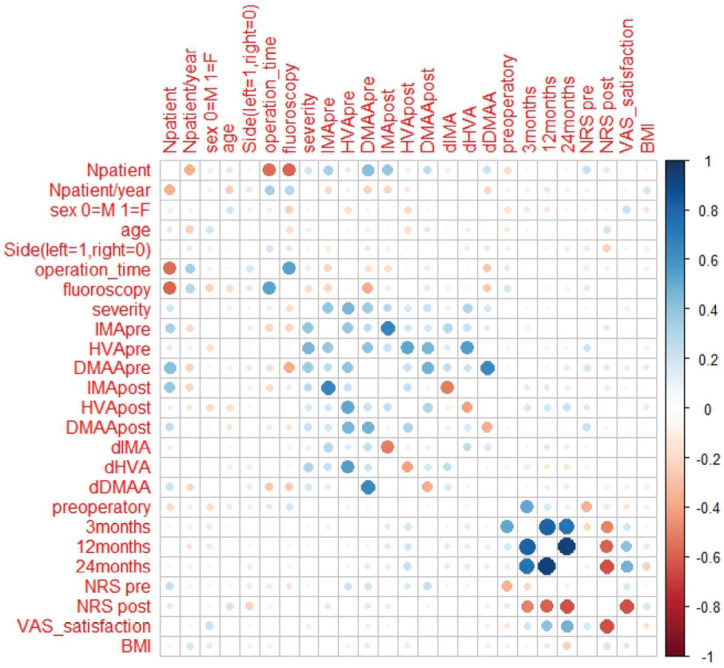
Correlation plot between number of patients and demographic, radiological and clinical data. Positive correlations are displayed in blue and negative correlations in red. Color intensity and size of the circles are proportional to the correlation coefficients.

Spearman’s rank correlation test was performed to assess correlations among all variables and patient order. There was no correlation between patient order and satisfaction (*p* = 0.87), suggesting that the performance of the surgeon was overall good according to the patients independent from their arrival order and changed only in terms of surgical time required (Figure 3).

A positive correlation between the operation time and fluoroscopy time of patients was detected (r = 0.51, *p* < 0.001). Operation time and fluoroscopy time were negatively correlated with patient order (r = −0.60, *p* < 0.001 and r = −0.61, *p* < 0.001, respectively), and thus the learning curve was focused on these variables.

**Figure 4:**
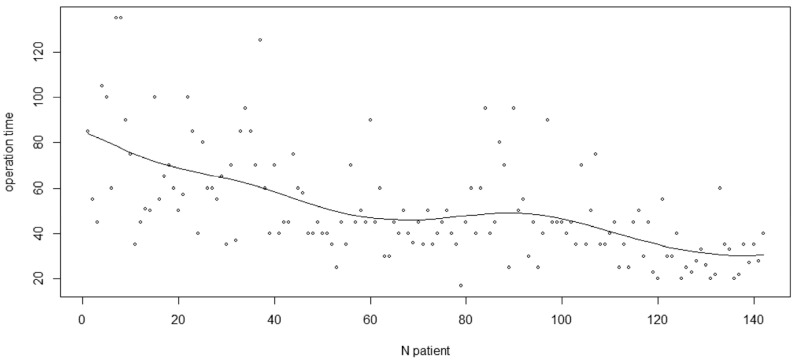
Non-parametric regression estimate of the operation time as a function of the number of patients treated (learning curve).

In Figure 4, the non-parametric estimation of the learning curve (operation time as a function of the number of patients treated) of the surgeon is reported [30]. Considering the period corresponding to the last twenty to thirty patients, it seems that the performance of the surgeon has stabilized.

We characterized the learning process as a sequence of periods with distinct characteristics, where the endpoints of each period were determined using a data-driven approach. Specifically, we employed a Bayesian change point model, which allowed us to model a sequence of data with different characteristics while simultaneously establishing the time windows when changes occurred. By examining the estimates of the last detected periods, we can infer whether a stabilization phase exists at the end of the learning process.

For this model, the posterior distribution summaries are reported in Table 2.

The most plausible values for the endpoints were identified after the treatment of the 9th and 112th patients, identifying three distinct periods: phase 1 (from 1 to 9 patients), phase 2 (from 10 to 112 patients) and phase 3 (from 113 to 142 patients). The estimated variability of the surgical time was higher in the first period (patients 1 to 9) with an estimated variance $\sigma^{21}=37$. This variability decreased significantly in subsequent periods, ($\sigma^{22}=18$ and $\sigma^{23}=10$ in the second and third periods, respectively). This is an indication that the surgeon’s performance tends to stabilize.

The estimated learning rate can be considered negligible in the first period, as the credible interval for the slope of the learning curve contains the value zero. In the second period (patients 10 to 112), operation time decreased on average by 0.08 min per additional patient (corresponding to an improvement of approximately 5 s per patient, with a credible interval of −0.23, 0.08). In the third period (patients 113 to 142), further performance gains were minimal. The point estimate is −0.23, but the large credible interval (−0.86, 0.68) centered on the value zero suggested substantial improvement, indicating that a plateau in the learning rate was reached. This means that the surgeon in the third period has terminated the learning–improving phase. The estimated mean operation times at the beginning of the three periods were 55.55, 43.92 and 27.95 min, respectively. Additionally, the estimated variance in the third period (10.90) was approximately 1/4 of the variance in the first period (37.16) and 1/2 the variance in the second period (18.16), providing further evidence of performance stabilization. These findings confirm that the learning phase concluded in the third period, marking the transition to a stable level of surgical proficiency. Within the model, we took into account the fluoroscopy time as a predictor, and we verified that indeed it is positively associated with the operational time.

The major complications included three cases of recurrence and one case of severe stiffness (ROM < 30°). The minor complications were slight loss of normal range of MTP joint motion (ROM 30–74°) in 20 cases. Five patients presented delayed wound healing, which healed completely in 4 weeks and did not require subsequent surgery. One patient complained of dysesthesia of the skin distal to the interphalangeal joint of the big toe because of neuritis of a cutaneous sensory dorsal branch, resolved spontaneously by the final follow-up. No cases of hallux varus due to overcorrection, malunion, delayed union or non-union were recorded. There were no cases of thrombo-embolism, no cutaneous or deep infections nor avascular necrosis of the metatarsal head. No case of dorsal displacement of the metatarsal head was recorded in this study.

Complication analysis showed that patients requiring a re-operation by phase of the learning curve were 1/9 (11.1%) in phase 1, 3/102 (2.94%) patients in phase 2 and 0/29 (0%) in phase 3. The overall complications reported by phase were 3/10 (30%) in phase 1, 20/110 (18%) in phase 2 and 1/22 (4.5%) in phase 3.

## 4. Discussion

The essential and innovative element in our analysis regarding RIAOs is the change point model used to thoroughly describe a learning curve study. It proved to be a useful tool for a correct interpretation of the learning curve, which was divided into three phases by the identification of two change points ($\tau^{1}\;\mathrm{and}\;\tau^{2}$). In phase 1, the improvement was not of particular interest, suggesting that the surgeon was initially approaching the technique. In phase 2, there was an estimated improvement of 8 min, while in phase 3, the estimated improvement was 21 min, indicating that these phases correspond to the learning phases. Importantly, the surgeon reached the plateau phase, showing slight variability and pointing out that the operator was consistently precise and effective. A similar trend was observed regarding the fluoroscopy time: the greatest improvements were recorded in phases 2 and 3 Based on these data, it can be supposed that the learning curve for RIAOs has a three-step learning curve to reach a plateau.

To the best of our knowledge, there is no study about a learning curve analysis on this surgical technique or any other minimally invasive or percutaneous procedure. Additionally, no learning curve has been performed using a Bayesian change point model, making the comparison with other learning curves on HV surgical techniques difficult. Toepfer et al. underlined the scarcity of literature on the learning curve of any surgical procedure for HV correction, especially when compared to other established orthopedic elective procedures such as MIS total hip replacement [5]. However, in the last years, several studies have been published regarding the learning curve of other MIS techniques, most of which involve different generations of the Minimally Invasive Chevron Akin osteotomy (MICA) procedure. In these studies, a plateau was reached after a mean of 35.5 cases; in particular, Palmanovich [31] reached a plateau after completing 27 cases, while Lewis [3] reached a plateau after 39 patients and Toepfer at 40 patients [5]. In our study, the real plateau was reached at 112 cases, as shown before, but the learning phase was divided by the change point model into different phases, with a steep initial learning phase of 9 patients (phase 1) and a smooth learning phase between 10 and 112 patients (phase 2), with an improvement of surgical time and fluoroscopy time, as highlighted in the previous paragraph in the second phase compared to the first (surgical time from 55 min to 43 min).

Although the plateau was reached earlier in the studies mentioned above, the studies were focused on different surgical techniques than in our study, and basic statistical analyses were employed. These aspects might have influenced the results. Specifically, analyzing surgical time, fluoroscopy time, complications and outcome in the same recent literature review [32], three studies [3,5,31] reported an average of 116.2 fluoroscopy seconds (range of 30–250) and a mean operating room time of 58.7 min (range of 31–185), both higher times to our phase 1 and 2 mean times.

Regarding clinical aspects, the reoperation rate ranged in the learning phase 6–12% and in the plateau phase 3–4%, and the complication rate ranged in the learning phase 16–16.67% and in the plateau phase 3.3–17% [19,33]. These results are comparable to the reoperation rate and complication rate in our learning curve. Considering the three phases in our study, there was an overall reduction in reoperation rate from 10 to 0% and a reduction in complication rate from 30 to 4.5%, and most of the complications were minor.

The MICA technique achieved an 87% satisfaction rate and a significant improvement in the AOFAS score [19]. Regarding the RIAOs, Bauer et al. [34] reported good results at 2 years of follow-up with a similar increase in the AOFAS score from 49 to 87.5 and an 89% satisfaction rate. In 2016, similar clinical outcomes were reported in a prospective study (the mean VAS score was 8.35/10 at the 24-month-follow-up period) [14], including 80 patients treated with the same technique at the final follow-up of 48 months, and the last radiographic assessments showed a statistically significant improvement compared with preoperative values. Only the results obtained in the correction of the severe HV deformities were less encouraging [35,36]. Finally, the technique caused some complications, including shortening and non-articular congruence and stiffness.

In agreement with these previous studies [14,19] and in line with the improvement of surgical outcomes achieved with the progression of the learning curve, VAS for patient satisfaction was 7.5 ± 1.9 at the last follow-up in our series. Further studies on RIAO, with a longer follow-up, could bring a better comparison with other percutaneous techniques for HV correction.

Similarly to other papers [5,6,7,37], no statistically significant differences were recorded in our analysis between the postoperative angles measured in the learning phases and the angles measured in the plateau phase, although there was a trend of improvement. These results can be explained by the fact that the surgeon tried to obtain the best correction and the best precision in every case. The correction of the angles did not show a variation during the learning curve. The AOFAS Scale showed a statistically significant improvement (*p* < 0.001) in the postoperative follow-ups. The NRS also showed a statistically significant improvement at the last follow-up (*p* < 0.001). Further, there was no statistically significant difference in the three phases of the learning curve. The accuracy of the technique was stable over time, while operating time and fluoroscopy time significantly improved. In a recent review [32] on learning curves in MIS for HV, an analysis of complications and outcomes showed similar results to our learning curve [5,6,7,19]. Some authors showed higher complication rates in the learning phase and lower reoperation rates in the plateau phase [19]; other authors showed better satisfaction rates, better HV angles and inter-metatarsal angle correction in the plateau phase [6,7]. In all papers, there was no statistically significant difference in complication rate and outcome between the learning phase 1 and the plateau phase [5,6,7,19].

Dr. Saltzman said that “benchmarks of proficiency in a surgical procedure should relate to the ability to consistently improve patients’ physical function and quality of life, not how many X-rays the surgeon takes in an operating room or how fast they can perform the surgery” [38]. For this reason, our analysis includes functional, clinical and radiographic outcomes and the personal improvement of a single surgeon performing MIS, which should be assessed during the learning curve.

A recent systematic literature review and meta-analysis indicate that a surgeon’s technical skill is a significant predictor of clinical outcomes. However, despite the development and validation of numerous scoring instruments to assess surgeon technical skills, there are surprisingly few articles that evaluate the association between skill and outcomes [39]. Hence, our original analysis aims to fill this gap by combining the evaluation of proper surgical technical parameters with the clinical and radiographic outcomes of the treated patients.

Two main aspects give originality and strength to our study: this is the first study in the literature on the learning curve of this procedure as well as a robust statistical analysis. This is the first time that the Bayesian model is used for a learning curve. In contrast to other recently published studies on learning curves about different HV surgical techniques that used basic statistical analysis, we assessed the learning curve and identified a possible plateau using a flexible statistical model. We emphasize that splitting the surgeon’s learning history into two or three periods arbitrarily [6,37] for the purpose of maximizing the difference in learning rate [40] does not automatically imply that a plateau is reached. With our study, we also found that the learning curve can be characterized by different average operation times but also different variability among the periods, with lower variance meaning stability in the proficiency of the surgeon. With the statistical model, we were able to consider covariates that possibly affect operation time, such as fluoroscopy time, which also reflects the complexity of the operation, thus adjusting for this possible patient-specific confounder and, finally, providing uncertainty quantification on learning rates and subperiod endpoints, which is not possible with simpler techniques [6,7,37,40].

The main limitation of this single-center case series study is that the sample was collected over a 10-year time with different rates of surgeries per year. However, each surgery was performed only on the first ray with no other associated procedures on the lateral rays during the follow-up time after proper but limited indications for mild-to-moderate symptomatic HV. Further, the only surgeon who performed the surgeries became an expert foot and ankle surgeon by the end of the study, so the results might not be generalizable to surgeons without experience in open surgical techniques or minimally invasive techniques for HV correction. The use of the AOFAS score for outcome measure was the most widespread health measurement in foot and ankle clinical practice when the study project was approved, but it is only partly validated [41] and may have overlooked some clinical aspects such as psychological ones. For these reasons, VAS for patient satisfaction was also used in this study.

Finally, selection bias is always possible because patients with indications for the MIS technique were patients with mild or moderate HV, so results are not generalizable for all deformities.

## 5. Conclusions

In this study, first, the learning curve analysis of the percutaneous Reverdin–Isham and Akin osteotomy in association with lateral release for HV correction was, for the first time, performed with a solid statistical method (a Bayesian model) to show how properly, not arbitrarily, the learning curve presented 3 phases:

The initial phase: “approaching the technique”.

The learning phase: “improvement”.

The plateau phase: “surgical accuracy and effectiveness”.

Second, this single surgeon’s learning curve showed an improvement in the surgical time and a reduction in fluoroscopy time already in the second phase, with further enhancement in the third phase where the true plateau phase of the curve was statistically found. In this way, good radiographic and clinical outcomes were achieved with patient satisfaction and low complication and reoperation rates.

Third, according to our initial hypothesis, an improvement in the various parameters evaluated was confirmed by this study during the learning process. Hence, the authors strongly believe its indication for the mild-to-moderate HV correction is still current.

## Figures and Tables

**Table 1:** Radiographic angles and pain.

	Baseline (Mean [±SD])	Follow-Up (Mean [±SD])	*t*-Test	*p*-Value
IMA	13.2 [2.4]	10.3 [2.3]	10.6	<0.001
HVA	26.5 [6.4]	13.2 [5.9]	18.3	<0.001
DMAA	11.9 [4.5]	5.8 [3.9]	12.1	<0.001
NRS	6.8 [1.3]	1.3 [1.3]	35.3	<0.001

Hallux valgus angle (HVA), inter-metatarsal angle (IMA), distal metatarsal articular angle (DMAA), Numerical Rating Scale (NRS), standard deviation (SD).

**Table 2:** Estimated parameters and 95% credible intervals for the change point model.

Posterior Summaries	5%	95%	Mean	Median
$\tau_{1}$	8.00	13.00	9.61	9.00
$\tau_{2}$	101.00	126.00	112.16	109.00
$\beta_{0}^{1}$	16.06	94.70	55.55	55.22
$\beta_{0}^{2}$	31.59	56.55	43.92	43.97
$\beta_{0}^{3}$	14.57	40.31	27.95	28.44
$\gamma^{1}$	−3.70	10.18	3.09	3.14
$\gamma^{2}$	−0.23	0.08	−0.08	−0.08
$\gamma^{3}$	−0.86	0.68	−0.21	−0.25
$\beta_{\mathrm{fluoroscopy}\;\mathrm{time}}$	0.14	0.43	0.30	0.30
$\sigma^{21}$	17.94	63.22	37.28	33.62
$\sigma^{22}$	15.50	20.93	18.16	18.06
$\sigma^{23}$	2.97	14.50	10.90	10.68

## Data Availability

The datasets used and/or analyzed during the current study are available from the corresponding author on reasonable request.
